# Hereditary paraganglioma presenting with atypical symptoms: Case report

**DOI:** 10.1097/MD.0000000000027888

**Published:** 2021-11-19

**Authors:** Shu Eguchi, Rintaro Ono, Takeshi Sato, Keigo Yada, Naoki Umehara, Satoshi Narumi, Yosuke Ichihashi, Taiki Nozaki, Naoki Kanomata, Tomonobu Hasegawa, Miwa Ozawa, Daisuke Hasegawa

**Affiliations:** a Department of Pediatrics, St. Luke's International Hospital, Tokyo, Japan; b Department of Pediatrics, Keio University School of Medicine, Tokyo, Japan; c Department of Pediatric Surgery, St. Luke's International Hospital, Tokyo, Japan; d Department of Molecular Endocrinology, National Research Institute for Child Health and Development, Tokyo, Japan; e Department of Diagnostic Radiology, St. Luke's International Hospital, Tokyo, Japan; f Department of Pathology, St. Luke's International Hospital, Tokyo, Japan.

**Keywords:** case report, paraganglioma, polyuria, posterior reversible encephalopathy syndrome, proteinuria, weight loss

## Abstract

**Rationale:**

Paraganglioma (PGL), an extra-adrenal pheochromocytoma, is a rare tumor, especially in children. While hypersecretion of catecholamines causes the classic triad of headaches, palpitations, and profuse sweating, prompt diagnosis is still challenging.

**Patient concerns:**

For 7 months, an 8-year-old boy complained of polyuria and weight loss, followed by proteinuria and headache for 1 month prior to admission. He was admitted to our hospital due to an afebrile seizure.

**Diagnosis:**

His blood pressure remained markedly elevated even after cessation of the convulsion. Magnetic resonance imaging of the brain revealed posterior reversible encephalopathy syndrome. Abdominal computed tomography showed a mass lesion encasing the left renal artery, measuring 41 mm in length along its major axis. The plasma and urine levels of normetanephrine were elevated. Additionally, iodine-123-metaiodobenzylguanidine scintigraphy showed an abnormal uptake in the abdominal mass with no evidence of metastasis. Based on these findings, we tentatively diagnosed him with PGL.

**Intervention:**

Substantial alpha- and beta-blocking procedures were performed, followed by a tumor resection and an extended left nephrectomy on day 31 of hospitalization. Pathological findings confirmed the diagnosis of PGL.

**Outcome:**

The postoperative course was uneventful, and his blood pressure normalized without the use of antihypertensive agents. Genetic testing revealed a known *SDHB* germline mutation. The same mutation was also detected on his father and paternal grandfather without any history of hypertension or malignant tumor.

**Lesson:**

It remains challenging to diagnose pheochromocytoma/paraganglioma (PPGL) promptly because PPGL can present with a variety of symptoms. Preceding symptoms of the presented case might be caused by PGL. Although PPGL is a rare disease, especially in children, it should be considered in differential diagnosis when various unexplained symptoms persist.

## 1 Introduction

Paraganglioma (PGL), an extra-adrenal pheochromocytoma, is a rare tumor. The incidence of pheochromocytoma/paraganglioma (PPGL) in adults is 2 to 5 per million, and the incidence in children is even lower.^[1]^ Approximately 30% to 40% of PPGL cases are hereditary, and pediatric cases are more likely to have germline mutations.^[2–4]^ While the hypersecretion of catecholamines causes the classic triad of headaches, palpitations, and excessive sweating,^[5–7]^ prompt diagnosis is still challenging. Here, we present a case of hereditary PGL with atypical initial symptoms.

## 2 Case presentation

**Figure 1:**
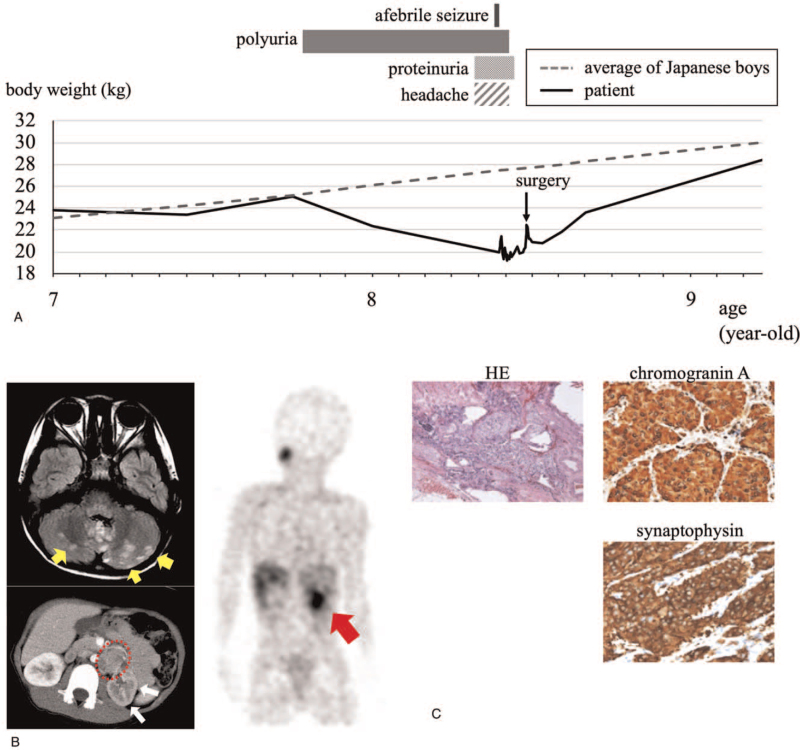
(A) The trend of the patient's body weight and the course of each symptom are shown with the average body weight of a Japanese boy. (B) Upper left panel: The magnetic resonance imaging (MRI) of the patient's brain on admission is shown. Axial fluid-attenuated inversion recovery MRI shows edema in the bilateral cerebellar hemispheres (yellow arrows). Lower left panel: The abdominal contrast-enhanced computed tomography on admission shows a partially ill-defined hypervascular mass, measuring 35 × 30 × 41 mm, at the left retroperitoneal space below the renal artery bifurcation (circle). Noted is a left kidney with atrophy and poor enhancement (white arrows). Right panel: Iodine-123-metaiodobenzylguanidine scintigraphy shows abnormal uptake in the abdominal mass (red arrow). (C) The results of hematoxylin and eosin staining, as well as the immunohistochemical results for chromogranin A and synaptophysin, are shown.

An 8-year-old boy, who required developmental follow-up at our hospital for developmental disorders, showed polyuria and weight loss 7 months before his admission. One month prior to admission, severe headache and proteinuria were also observed (Fig. 1A). On the day of admission, the patient suffered generalized convulsion and was transported to our hospital. Although his convulsions stopped spontaneously within 2 minutes, his impaired consciousness persisted, and his blood pressure was elevated at 180/140 mm Hg. A physical examination revealed no abnormal findings, and brain magnetic resonance imaging showed high signal intensity in the bilateral cerebellar hemispheres on fluid-attenuated inversion recovery, suggestive of posterior reversible encephalopathy syndrome (Fig. 1B, upper left panel). Abdominal contrast-enhanced computed tomography revealed a partially ill-defined hypervascular mass with heterogeneous enhancement, measuring 35 × 30 × 41 mm, at the left retroperitoneal space below the renal artery bifurcation. A necrotic area was seen in the mass, and the left kidney was atrophic and poorly enhanced (Fig. 1B, lower left panel). The plasma and urine catecholamine levels were both elevated: plasma noradrenaline: 2.1 ng/mL (reference value: 0.10–0.50 ng/mL); urine noradrenaline: 330 μg/day (reference value: 31–160 μg/day); plasma free normetanephrine: 912 pg/mL (reference value: ≤506 pg/mL); and urine normetanephrine: 1.4 mg/day (reference value: 0.09–0.33 mg/day). Levels of adrenaline, dopamine, and metanephrine were within normal limits in both the plasma and the urine. Iodine-123-metaiodobenzylguanidine scintigraphy showed abnormal uptake in the abdominal mass, and there was no evidence of metastasis (Fig. 1B, right panel). With the clinical diagnosis of noradrenaline-producing PGL, we initiated antihypertensive therapy using a calcium-channel blocker and an alpha-blocker (doxazosin). Although a hypertensive attack with convulsion recurred on the seventh day of admission, the blood pressure was well-controlled, and the dose of doxazosin was gradually increased from the initial dose of 1 mg thereafter. After the dose of doxazosin was increased to 16 mg per day, a beta-blocker (carvedilol) was added for heart rate control. The patient underwent resection of the tumor on the 31st day of hospitalization. A left total nephrectomy was also performed because the tumor was tightly adhered to the left renal vessel, and the left kidney was highly atrophic. During the perioperative and postoperative periods, no adverse events, such as hypertensive or hypotensive attacks, occurred, and the patient was discharged on the 10th postoperative day. Immunohistochemical findings of the tumor cells revealed positivity for chromogranin A and synaptophysin, and the diagnosis of PGL was confirmed (Fig. 1C). The Grading of Adrenal Pheochromocytoma and Paraganglioma score was 4 (moderately differentiated type), and Ki67 was positive in 16% of the cells. The levels of noradrenaline and normetanephrine in both the plasma and the urine normalized after the surgery. All symptoms, including the hypertension, headache, polyuria, and proteinuria, disappeared, and the body weight started to increase. The patient remained asymptomatic without relapse, and his blood pressure remained stable without hypotensive agents for 11 months after the surgery. The genetic testing revealed a known heterozygous *SDHB* germline mutation (c.201-2A>C), which was also detected on his father and paternal grandfather without any history of hypertension or malignant tumor.

## 3 Discussion and conclusions

It remains challenging to diagnose PPGL promptly because PPGL can present with various symptoms due to hypersecretion of catecholamines. In addition to the classic triad of headaches, palpitations, and profuse sweating, other symptoms, such as tachycardia, hypertension, syncope, anxiety, tremor, hyperglycemia, and weight loss, are frequently observed.^[5–7]^ Erickson et al^[8]^ reported that hypertension, headache, perspiration, and palpitations were commonly presented, with the frequencies of 64%, 26%, 25%, and 21%, respectively. Although hypertension is often the first trigger to suspect PPGL, some cases show normal blood pressure in early stages of the disease.^[9]^ In addition, blood pressure is not routinely measured in a pediatric outpatient clinic. It is notable that uncommon symptoms of PPGL, such as polyuria^[10]^ and proteinuria,^[11]^ were observed in the presented case about half a year prior to development of the classic symptoms. Possible causes of polyuria might be a suppression of antidiuretic hormone due to catecholamines or increased sodium excretion from kidney induced by hypertension.^[10]^ Similarly, catecholamines and hypertension can cause renal impairment, which in turn results in proteinuria.^[11]^ Although PPGL is a rare disease, especially in children, it should be considered in differential diagnosis when various unexplained symptoms persist.

Although plasma and urine levels of catecholamines were considered as a standard diagnostic method for PPGL, elevated level of serum or urine metanephrines has been reported as more sensitive diagnostic marker.^[12,13]^ The mainstay of management for PPGL is surgical removal because PPGL is generally refractory to chemotherapy or radiotherapy. Both hypertension and hypotension are critical perioperative complications of PPGL. Hypertension with catecholamine surge might be caused by tumor manipulation, while hypotension could be caused by the abrupt cessation of catecholamine secretion after tumor removal.^[14]^ To reduce the risk of perioperative complications, thorough fluid volume correction and adequate blood pressure control with alpha-blockers has been reported to be quite important.^[15,16]^

According to previous reports from the United States and Europe, approximately 30% to 40% of PPGL cases are hereditary.^[2–4,17]^ These reports indicated that pediatric cases are more likely to have germline mutations, multiple site lesions, and higher risks of recurrence than adult cases. Jochmanova et al^[2]^ reported that 85% of children with PPGL had germline mutations, of which *SDHB* mutation was the most common (62%). Because 50% of pediatric patients with *SDHB* germline mutation will develop metastasis in 7 years from diagnosis, long-term survival rate decreased year by year: 10-, 20-, 30-, and 40-year survival rates were 97%, 78%, 44%, and 22%, respectively.^[2]^ Genetic testing should be considered for PPGL patients, especially in younger cases, and more intensive follow-up is needed regarding hereditary cases. Because there are so few reports of hereditary PPGL from Asia,^[18]^ the proportion of germline mutations in Asian patients with PPGL still needs to be clarified.

## Author contributions

**Conceptualization:** Shu Eguchi, Rintaro Ono, Daisuke Hasegawa.

**Funding acquisition:** Tomonobu Hasegawa.

**Investigation:** Takeshi Sato, Satoshi Narumi, Yosuke Ichihashi, Tomonobu Hasegawa.

**Resources:** Shu Eguchi, Rintaro Ono, Keigo Yada, Naoki Umehara, Miwa Ozawa, Daisuke Hasegawa.

**Writing – original draft:** Shu Eguchi, Rintaro Ono, Daisuke Hasegawa.

**Writing – review & editing:** Takeshi Sato, Satoshi Narumi, Taiki Nozaki, Naoki Kanomata.
